# The Use of Adrenalin Chlorid in Local Anesthesia

**Published:** 1904-10-15

**Authors:** B. B. Atchison

**Affiliations:** Elgin, Ills.


### THE USE OF ADRENALIN CHLORID IN LOCAL ANESTHESIA.

BY B. B. ATCHISON, D.D.S., ELGIN, ILLS.

Having used local anesthetics since beginning thepractice of dentistry, and being a strong advocate of their use to a certain extent, I have cast about in the domain of drugs at the disposal of the dentist for the safest and most desirable and, at the same time, the most efficient agent of that kind.

Having seen and read of the disastrous results of the employment of the various preparations of cocain on the market, I entered upon the use of any solution in which it was incorporated with the greatest caution, for we all know that cocain cannot he handled with too much care, though willing to admit what a blessing to mankind it is in the relief of pain in minor surgery, when used with the proper precautions.

During my first year in practice, after much thought, I had my druggist prepare for me a solution of cocain, nine grains; antipyrin, two grains; distilled water, one ounce, two percent solution. This I used with very gratifying results for at least four years with scarcely a case of dangerous symptoms. However, I might say that I was always careful to have a fresh solution on hand, at least a fresh solution was prepared every seven to ten days, as at the end of or near that time a fungous growth would begin to accumulate, when I deemed the solution unsafe. But yet, having used this formula so long, I could not help notice in certain patients the unmistakable symptoms of the physiologic effects of cocain. As a matter of caution I had very nearly given up the use of the drug altogether, when one day my attention was directed to the possibilities of adrenalin chloride, prepared by Parke, Davis & Co., in which I became very much interested, as I learned the physiologic action of the drug, etc. The idea occurred to me that adrenalin would make a happy combination with my local anesthetic, and I proceeded to experiment with the combination. I found that by adding one fluidrachm of adrenalin to the above formula, I could safely use it in all cases, as the adrenalin, being a vaso-constrictor, aided greatly in rapidly anesthetizing tissue locally. It retards the absorption of the cocain so that anesthesia may be produced with about just half the quantity of cocain formerly used. It also greatly alleviates the danger of cocain, not only by reducing the amount necessary, but also by virtue of its action as a cardiac stimulant.

